# Monthly Summary

**Published:** 1882-10

**Authors:** 


### MONTHLY SUMMARY.

---

*The use of Tobacco by Boys.*—The use of tobacco by growing boys is so generally recognized as pernicious, that it is extraordinary that more energetic measures are not urged upon those having the care of the youth to prevent the habit. Already it has been prohitited in the U. S. Naval Academy, at Annapolis, in the U, S. Military Academy, at West Point, in the Phillips Exeter Academy, New Hampshire, and in various other enlightened educational institutions.

This was not the result of prejudice or hobbyism. If any set of men are free from these vices of learning, it is the naval surgeons. and it was especially from them, and particularly from Dr. A. L. Gihon, U. S. N., that this attack on the weed began. The indictment laid against it charged :—

1. That it leads to impaired nutrition of the nerve centres.

2. That it is a fertile cause of neuralgia, vertigo and indigestion.

3 That it irritates the mouth and throat, and thus destroys the purity of the voice.

4. That, by excitation of the optic nerve, it produces amaurosis and other defects of vision.

5. That it causes a tremulous hand and an intermittent pulse.

6. That one of its conspicuous effects is to develop irritability of the heart.

7. That it retards the cell change on which the development of the adolescent depends.

This is a formidable bill of particulars, and yet each of these charges is preferred by the best modern authority, and what is more, each is substantiated by an abundance of clinical evidence

Testimony is also adduced from the class records of schools and colleges, which indicate very positively that the effect of tobacco on the mental faculties is deteriorating. The best scholars are not tobacco users; non-smokers take the highest rank in every grade; and whether we look at the exceptionally brilliant students, or compare the average of those who use and those who refrain from tobacco, the result shows the same.

With these facts staring us in the face, it becomes the duty of every schoolmaster and every parent to set himself resolutely against the beginning of this injurious indulgence.

It is, indeed, no easy matter to prohibit it successfully. There is a curious attraction about this nauseous plant which has never been explained. A habitual consumer of it cannot explain its fascination. It has extended over the world with marvelous facility.

Nevertheless, we believe that the youth of America are intelligent and ambitious enough, in the aggregate, to be trusted. If the conseqnences of tobacco using are plainly stated by an authority that a lad respects, it will often lead him to drop the habit, or to refrain from beginning it, when threats and punishments would not. The latter he regards as an exercise of arbitrary power, the former appeals to his reason and good sense. It is the duty of a physician to express himself plainly on this subject, and he can only do so by condemning the habit in boys, at any rate.—*Editorial in Medical and Surgical Reporter.*

---

*Scurvy and Fresh Meat.*—In commencing upon the report of Dr. W. H. Neale. Medical Officer of the Arctic steamer *Eira*, the *Lancet* says:—

The most interesting of Mr. Neale's observations, however, is that which relates to the absence of any symptoms of scurvy among the men—a fact which has led him to express the opinion that if men live on the flesh of animals indigenous to the country, even without vegetables, they will run very little risk of scurvy, so that, under such circumstances, lime juice is not of much use. Curiously enough, while Mr. Neale is detailing his experience with regard to the prophylactic value of fresh meat against scurvy in the arctic region, Dr. Lucas write to us from India, stating that the meat eating tribes of the northwest provincs are comparatively free from scurvy, while the vegetable-feeding tribes are not unfrequently attacked with the disease. This experience of both arctic and tropical observers, which does not stand alone, is so entirely distinct from European experience, that some solution of the apparent paradox is required. In a letter which we published some time since, a correspondent points out that the statements of Mr. Neale and Dr. Lucas need not be considered as in any way upseting our established views with regard to the disease, since, as he urges, meat is probably a scorbutic and an antiscorbutic article of diet, according to the period of time that elapses from the time of slaughter to the period of cooking. Fresh muscle, as is well known, has an alkaline reaction, due to the presence of the neutral sodium phosphate ; after rigor mortis has passed off the reaction becomes acid, due to the development of lactic acids; the neutral phosphate is thus converted into acid sodium phosphate. In hot countries the meat is eaten so freshly killed that lactic acid is not developed ; in arctic regions the cold stops its formation; in European countries, where meat is usually hung, there is ample time for its generation. Thus in tropical and arctic regions the muscle plasma is alkaline when cooked, in European countries acid. If, therefore, it be true that scurvy is produced by a diminution of the alkalinity of the blood—a view originally put forward by Garrod and subsequently confirmed and extended by Dr. Ralfe—then we can conceive how flesh meat may be antiscorbutic, while hung meat will have an opposite quality. Lastly, Mr. Neale is to be thanked for his suggestion of the use of blood as an anti-scorbutic. If its employment on future occasions should further prove its prophylactic value with regard to scurvy, we shall expect to see it extensively use by our mercantile marine, while under any circumstances, it introduces to the notice of travelers and voyagers a food at once portable, nutritious, and wholesome.

---

*Ether.*—In the *Lancet*, Dr. H. Bendelack Hewetson discusses the relative value of ether when prepared with "rectified" or "methylated" spirits of wine. The author state that there is a great difference between them. That prepared with rectified spirits has been found by those who have used it less safe and less desirable, producing more sickness and laryngeal spasm in certain cases in which there is a tendency to such complications.

Of the use and applicability of the methylated ether—as the safest anæsthetic known, when carefully administered by means of Clover's inhaler—he can speak strongly, as the result of daily observation. It is a very ordinary circumstance to occupy eighty seconds in producing complete anæsthesia. without a struggle or a cough, and it is by no means extraordinary for a patient to be "fully under" within the minute. In the case of short operations upon the eyes, and the like, it is hardly ever necessary to re-apply the inhaler after it has been once removed for the operator to commence, the patient remaining sufficiently anæsthetic for an operation such as mentioned to be completed without hurry. Anæsthesia can be prolonged with equal safty even so far as to keep a patient in labor completely under its influence for upward of *four hours* the longest time which has happened in my experience. Methylated ether is, I consider, from this point of view, the safest and cheapest anæsthetic at present in use.—*Medical and Surgical Reporter.*

---

*Microscopic Structure of Malleable Metals.*—The following observations on the minute structure of metals which have been hammered into thin leaves are instructive. Notwithstanding the great opacity of metals, says *Manufacturer and Builder*, it is quite possible to procure, by chemical means, metallic leaves sufficiently thin to examine beneath the microscope by transmitted light. Such an examination will show two principal types of structure, one essentially granular and the other fibrous. The granular metals, of which tin may be taken as an example, present the appearance of exceedingly minute grains, each being perfectly isolated from its neighbors by still smaller interspaces. The cohesion of such leaves is very small.

The fibrous metals, on the other hand, such as silver and gold have a very marked structure. Silver especially has the appearance of a mass of fine, elongated fibers, which are matted and interlaced in a manner which very much resembles felt. In gold, this fibrous structure, although present, is far less marked. The influence of extreme pressure upon gold and silver seems to be therefore, to develop a definite internal structure. Gold and silver, in fact, appear to behave in some respects like plastic bodies. When forced to spread out in the direction of least resistance their molecules do not move uniformly, but neighboring molecules, having different velocities, glide over one another, causing a pronounced arrangement of particles in straight lines.

---

*No Salivation from Amalgam Filling.*—I have practiced dentistry thirty-nine years. I never saw a case of salivation produced by amalgam fillings.

For the last eight year I have used amalgams constantly, amounting to eight hundred amalgam fillings per pear, for the *average* of those eight years. I have mixed the amalgam in the palm of my left hand for more than nine-tenths of that. I have never had any symptom of the pathological effects of mercury, either local or general. On the contrary, I have had better health than for ten years previous, and the color of my face has changed from paleness to healthy, ruddy glow, and that, too, without the help of "beer, wines, or spirits."

All well read physiologists know that *experiments* have demonstrated that *very* small quantities of mercury increase the number of *red* blood corpuscles.—*Henry S. Chase, M. D., D. D. S.*

---

*Cement for Rubber.*—Powdered shellac is softened in ten times its weight of stong water of ammonia, whereby a transparent mass is obtained, which becomes fluid after keeping some little time without the use of hot water. In three or four weeks the mixture is perfectly liquid, and, when applied, it will be found to soften the rubber. As soon as the ammonia evaporates the rubber hardens again—it is said quite firmly—and thus becomes impervious both to gases and to liquids. For cementing sheet rubber or rubber material in any shape, to metal, glass and other smooth surfaces, the cement is highly recommended.

---

*Crural Neuralgia Among Dentists.*—On June 20th I was called to a gentleman said to be suffering severely from sciatica. The patient was resting on his right side, complaining of intense pain in the left loin, radiating thence along the outer and anterior aspect of the left thigh whenever he attempted to move. Firm pressure, applied between the tuberosity of ischium and great trochanter of femur was painless, but the instant one touched the skin immediately over the erector spinæ muscle severe pain was evoked, extending down the thigh to the knee-joint, mapping out exactly the course of the anterior, crural and external cutaneous nerves. Pressure over the points of exit of the second, third and fourth lumbar nerves from the spinal canal caused excessive pain. The nerves at fault were clearly the second, third and fourth lumbar, the hyperæsthetic area in the loin clearly corresponding to the distribution of the posterior divisions of these nerve trunks. The case was obviously not one of sciatica, but crural neuralgia, having a very unusual distribution; the ordinary forms of this disease extend to the foot and toes of the affected leg. Belladonna and aconite were applied locally, trychnia internally; the patient was convalescent in seven days.

Two days later a similar case came under my notice, and a third has been reported to me with exactly similar symptoms. Curiously enough, the three patients were dentists, engaged in the active duties of their profession. It appears exceedingly probable that this painful affection may be explained by the fact that when dentists operate they always stand at the right side of the patient, consequently, when manipulating cavities in teeth difficult of access it is necessary to throw themselves into a constrained attitude, whereby the lumbar vertebræ are slightly flexed anteriorly, but flexed laterally to a considerable degree. It is necessary, sometimes, to maintain this cramped position for long periods, the temporary distortion being even more exaggerated when the dental engine is being used. These combined flexions cause their lumbar nerves to become congested, irritated and possibly injuriously nipped as they pass through intervertebral foramina, thus giving rise to the symptoms detailed above.—*Dr. J. B. Sutton in London Lancet.*

